# Response characterization of radiochromic OC-1 films in photon, electron, and proton beams

**DOI:** 10.1002/mp.17356

**Published:** 2024-08-26

**Authors:** Qinghao Chen, Xiandong Zhao, Jufri Setianegara, Yao Hao, Tianyu Zhao, Tiezhi Zhang, Arash Darafsheh

**Affiliations:** Department of Radiation Oncology, Washington University School of Medicine in St. Louis, St. Louis, Missouri, USA

**Keywords:** dosimetry, OC-1, optical density, OrthoChromic, radiochromic film

## Abstract

**Background:**

Radiochromic film (RCF) dosimeters with their high spatial resolution and tissue equivalent properties are conveniently used for two-dimensional and small-field dosimetry. OC-1 is a new model of RCF dosimeter that was commercially introduced recently. Due to its novelty there is a need to characterize its response in various radiation beam types.

**Purpose:**

To study the response of OC-1 RCFs to megavoltage clinical x-ray, electron, and proton beams, as well as kilovoltage x-ray beams used in a small animal research irradiator.

**Materials and methods:**

OC-1 RCFs were cut into ~4 × 4 cm^2^ pieces. RCF samples were irradiated at various dose levels in the range 0.5–120 Gy using different modalities; a small animal radiation research platform (SARRP) (220 kVp), a medical linear accelerator (6 MV, 10 MV, 15 MV, 6 MV FFF, 10 MV FFF photon beams, as well as 6 and 20 MeV electron beams), and a gantry-mounted proton therapy synchrocyclotron. In order to study any dependency on the fractionation scheme, same dose was delivered at several fractions to a set of films. Different dose rates in the range 200–600 MU/min were delivered to a set of films to investigate any dose rate dependency. The films were scanned preirradiation and at 48 h post-irradiation using a flatbed scanner. The net optical density (OD) was measured for red, green, and blue color channel for each film. The orientation dependency was studied by scanning the films at eight different orientations. In order to study the temporal evolution of the response of the films, film samples were irradiated at 10 and 50 Gy using 6 MV photon beams and were scanned upon irradiation at certain time intervals up to 3 months. The spectral response of the films were studied over the visible range using a spectrometer.

**Results:**

For megavoltage photon, electron, and plateau region of the proton beams, we did not observe a significant dependency on the beam quality, dose rate, and fractionation scheme. At the kV beam, an unusual over-response was observed in the films’ net OD. An orientation dependency in the response of the films with a sinusoidal trend was observed. The response of the films increased with time following a double or triple exponential trend. The spectral absorption peaks were blue-shifted with dose.

**Conclusion:**

OC-1 RCFs were found to be reliable dosimeters with no significant energy dependency in MV range for photon and electron beams including the FFF beams. They over-respond when irradiated by kV x-ray beams compared to MV x-ray beams. Caution must be exercised to maintain the orientation of the films when scanning. Due to the temporal growth in the net OD of the films, same post-irradiation time interval must be used for scanning the calibration and test films.

## 1 | INTRODUCTION

Radiochromic film (RCF) dosimeters are ubiquitous in medical physicists’ dosimetry arsenal thanks to their high resolution, ease-of-use, and water-equivalency, as well as their ability to provide two-dimensional (2D) dose distributions of the radiation fields.^1–3^ Their suitability for clinical or research use in external beam photon, electron, and ion therapies as well brachytherapy has been demonstrated by many investigators.^4–13^ On a fundamental level, working principle of RCF dosimeters is based on radiation-induced polymerization of their constituent monomers. Polymerization process leads to a visual change in color in the films that is quantified to measure the dose; the most common metric for such quantification is optical density (OD). An ad hoc calibration curve is established in advance by exposing RCFs to several known levels of dose and obtaining the OD versus dose relationship.

GafChromic films (Ashland Inc., Bridgewater, NJ, USA) are the most commonly commercially available RCFs. Different GafChromic film models are in the market to support applications involving various dose ranges.^4,5^ The main structural difference between these models is the size of the monomer nanocrystals in the active layer; the smaller they are, the less sensitive that model is. EBT-3 model with nominal dynamic range of 0.2–10 Gy is the most commonly used model for radiation therapy quality assurance.^14,15^ EBT-XD model has extended dynamic range (0.4–40 Gy) to cover SBRT/SRS dosimetry needs.^16–19^ Less commonly used RCF models in radiation therapy are the MD-V3 and HD-V2 models with dynamic range of 1–100 Gy and 10–1000 Gy, respectively.^20,21^ The active layer is symmetrically sandwiched between 125-μm thick matte polyester layers in EBT3, EBT-XD, and MD-V3 models. The HD model, however, is not symmetric and it only has a coating on one side (97-μm-thick clear polyester). The nominal thickness of the active layer is 28, 25, 15, and 8 μm in EBT3, EBT-XD, MD-V3, and HD-V2 models, respectively. The constituent monomer crystals in the active layer have different sizes in these models enabling them to cover different dose ranges.

In 2020, OrthoChrome Inc. (Hillsborough, NJ, USA) launched their RCF product under OrthoChromic film or OC-1 film name. The nominal dose range for OC-1 film is 0.1–100 Gy according to the manufacturer. The thickness of the sensitive layer of such films is ~30 μm coated over an approximately 125-μm-thick white polyester base. Due to their novelty, there is a need for independent study of the response of such films under different radiation fields.^22–24^

The objective of this work is to study the response of OC-1 films to clinical megavoltage photon (6 MV, 10 MV, 15 MV, 6 MV FFF, and 10 MV FFF), electron (6 and 20 MeV), and proton beams, as well as to a small animal irradiator’s kilovoltage x-ray beam (220 kVp). Dose-, dose-rate-, energy-, and orientation-dependency of the films as well as their spectral properties are studied. The effect of fractionation and temporal evolution of the response of the films are studied as well.

## 2 | MATERIALS AND METHODS

### 2.1 | Film preparation and analysis

OC-1 RCFs were cut in 3.8 cm × 3.8 cm pieces and marked in their corner to maintain orientation during pre- and post-irradiation scanning. The films were scanned in reflection mode using a flatbed scanner (Expression 10000XL, Epson Americana Inc., Long Beach, CA, USA) in TIFF format with RGB colors,48 bit per color channel, spatial resolution of 300 dpi, and all color corrections offered by the scanner’s software turned off. Films were placed at the center of the scanner in a reproducible manner. Films were handled according to the manufacturer’s and AAPM’s TG-235^2^ recommendations. The exposed films were scanned 48 h after irradiation to minimize time-dependent response of the film.

MATLAB (MathWorks, Natick, MA, USA) was used to obtain the pixel values, at each color channel, representing the reflected light intensity. Each film sample was scanned three consecutive times and the average was calculated and used for data analysis. The net OD (Net OD) of each film sample for each color channel was measured according to Equation (1), in which $P_{0}$ and $P_{f}$ are the pixel value of the scanned un-irradiated and irradiated film, respectively.

(1) $$\text{Net}\;OD={log}_{10}\frac{P_{0}}{P_{f}}$$

In order to derive the films’ sensitivity (S), dose–response curves for each color channel was obtained by fitting the Net OD values using Equation (2):

(2) $$\text{Net}\;OD=A\times D+B\times D^{m}$$

where A,B, and m are fitting parameters. The first derivative of Equation (2) with respect to dose (D) yields the films sensitivity.^25^

(3) $$S=A+m\times B\times D^{m-1}$$

A calibration curve for red, green, and blue color channels was established by fitting the Net OD data using Equation (4) in which a,b, and *n* are fitting parameters.

(4) $$D=a\times Net\;OD+b\times Net\;OD^{n}$$

**FIGURE 1:**
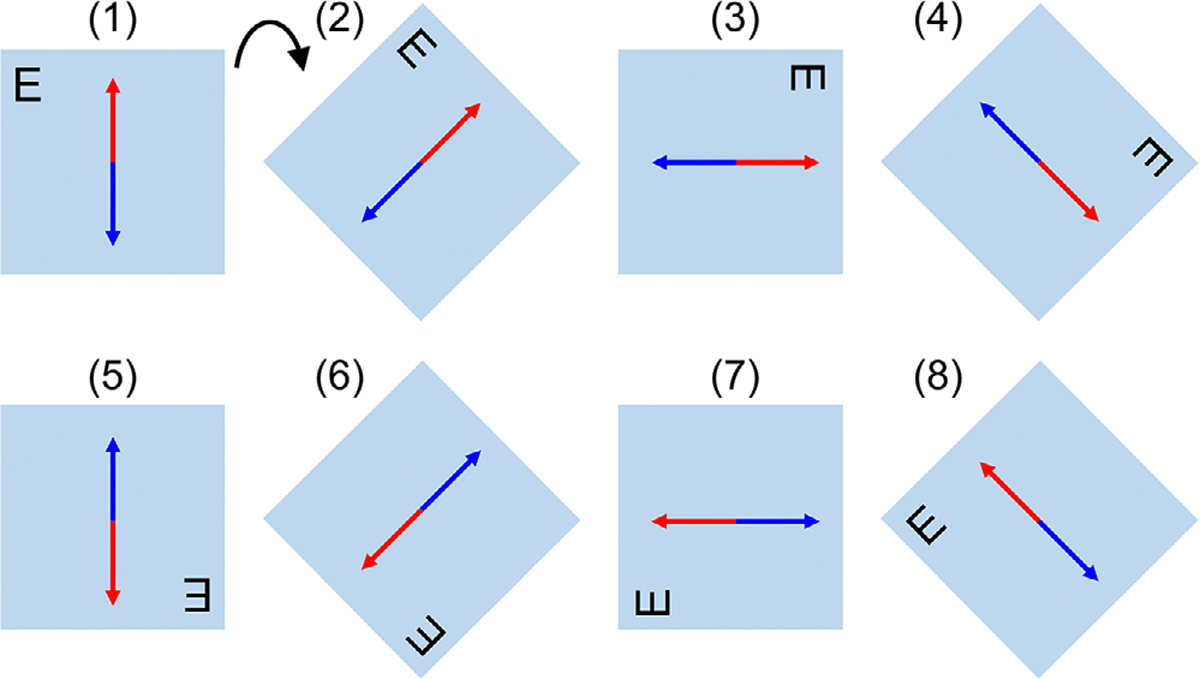
Eight different orientations of a film sample with respect to the flatbed scanner. The straight arrows represent an arbitrary hypothetical ensemble direction of the nanocrystals in the active layer of the film. Panels (2–8) were obtained by sequential 45° clockwise rotation of panel (1) around its center (see the curved arrow). Letter E indicates where the films have been marked.

In order to investigate the orientation dependency of the films, several irradiated and non-irradiated films were scanned in eight orientations as shown in Figure 1. Panel (1) shows the “reference” orientation used for all measurements. The “reference” orientation was chosen since the films were cut in landscape orientation, and it does not necessarily represnt a particular direction of polarization (the arrows in Figure 1 were only drawn to convey the concept of existance of a polarization axis). Panels (2–8) were obtained by consecutively rotating the film 45° clockwise around its center. This will allow us to observe in more detail to what extent the orientation of the film with respect to the light source would influence the OD of the film. The OD at any orientation was measured using the same setting used for other film samples.

### 2.2 | Dose response to different radiation types and beam qualities

The films were irradiated with 220 kVp, 6 MV, 6 MV FFF, 10 MV, 10 MV FFF, and 15 MV photon beams, 6 and 20 MeV electron beams, and a proton beam. For each beam quality, consistent dose levels were employed, specifically 0.5, 1, 2, 5, 10, 20, 50, 100, and 120 Gy. To prevent excessive strain on the machine, the proton beams did not utilize the 120 Gy dose level. It should be mentioned that although the maximum recommended dose for this film model is 100 Gy, we considered delivering up to 120 Gy to evaluate their response beyond the vendor’s recommended dynamic range. To mitigate uncertainties associated with individual films, three films were replicated for each beam quality and dose level. This approach aimed to minimize the impact of any potential variations and enhance the reliability of the obtained data.

**FIGURE 2:**
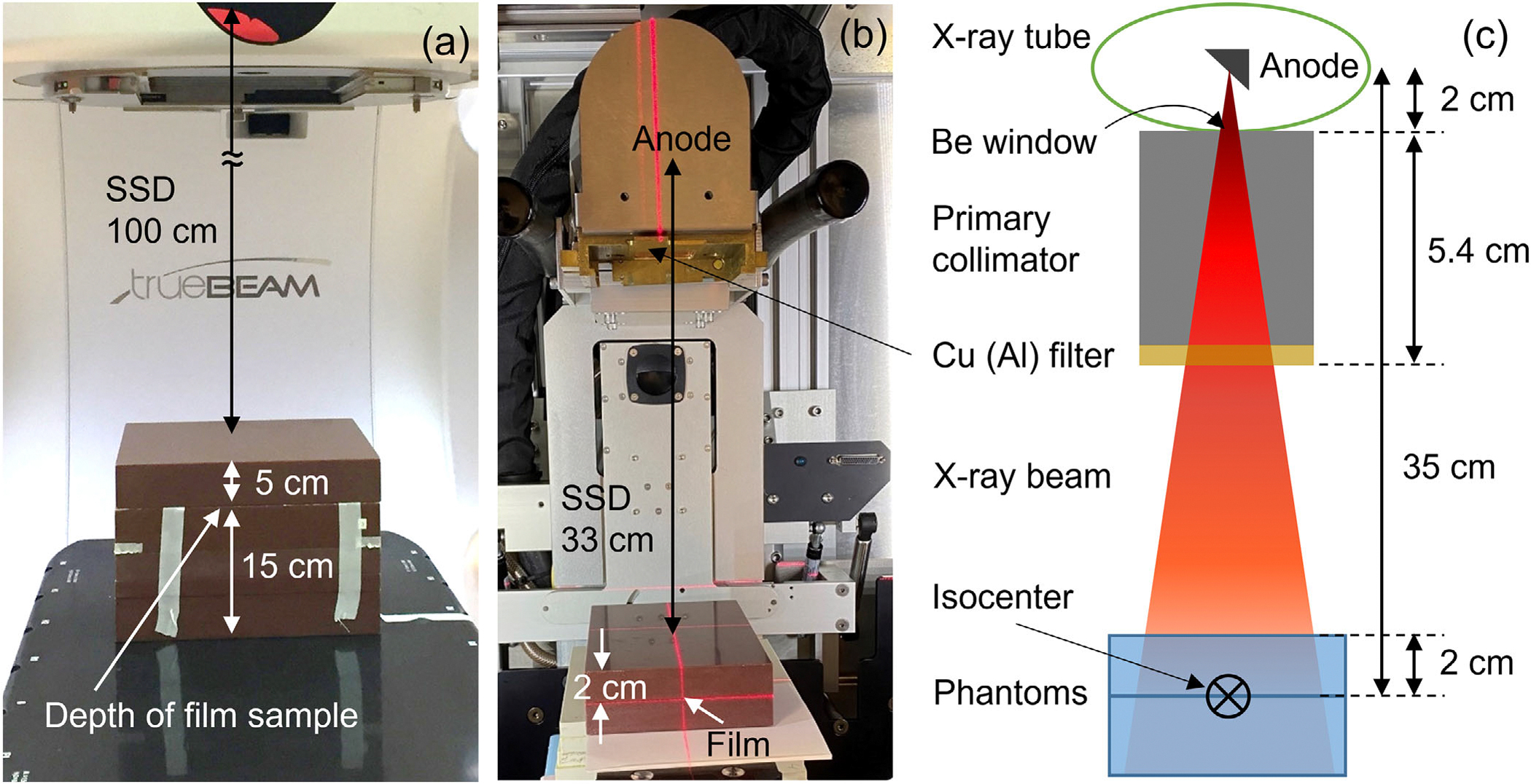
(a) Geometry of the film irradiation setup with a linac. Films were placed at 5 cm depth in phantom at SSD of 100 cm. (b, c) Geometry of the irradiation setup with the SARRP. Films were placed at 2 cm depth in phantom at SSD of 33 cm. Panel (c) drawn not to scale. SARRP, small animal radiation research platform; SSD, source-to-surface distance.

The megavoltage photon and electron beams (6 MV, 6 MV FFF, 10 MV, 10 MV FFF, and 15 MV) were generated by a linac (TrueBeam, Varian, Paolo Alto, CA). The linac had been calibrated according to AAPM’s TG-51^26^ to deliver 1 cGy/MU at the depth of maximum dose. For photon beams, the films were placed at 5 cm depth in solid water phantoms with additional 15 cm solid water phantom underneath them to generate sufficient backscatter. The source-to-surface distance (SSD) was 100 cm and the field size was 10 × 10 cm^2^. For electron beams, the films were positioned in solid water at the depth of maximum dose, specifically at 1.2 cm for 6 MeV beams and 2.7 cm for 20 MeV beams. The SSD was set at 100 cm, and a 15 × 15 cm^2^ electron applicator was used. The experimental setup of film irradiation with the linac is illustrated in Figure 2a.

The proton irradiation was performed using a pencil beam scanning system (Mevion Medical Systems, Littleton, MA). The films were placed at 5 cm depth in phantom and the calibration field consisted of 41 × 41 proton spots, each with 1 MU, scanning a 10 cm × 10 cm area was delivered. Protons had full energy (227 MeV). The machine had been calibrated according to TRS-398 protocol to deliver 1 Gy physical dose under the calibration condition.

Figure 2b, c present the geometry of the irradiation setup with the small animal radiation research platform (SARRP, Xstrahl GmbH, Germany). SARRP employs a rotating anode x-ray source. Nominal energy of the x-rays is 220 kVp at 13 mA current. The SARRP had been calibrated according to AAPM’s TG-61^27^; the calibrated dose rate at the isocenter was 3.67 Gy/min under standard condition (2 cm depth in phantom, 33 cm SSD and full scatter) with the 0.15 mm Cu filter without any additional secondary collimator set. In order to provide calibration curves, the films were placed at the isocenter (35 cm source-to-axis distance, SAD) at 2 cm depth in solid water phantoms with 5 cm additional phantom beyond them. The films underwent irradiation using the aforementioned dose levels. The deliveries involving greater or equal than 50 Gy dose were done at 25 Gy fractions with 10 min delay in between in order to avoid excessive pressure to the machine.

### 2.3 | Dependency on dose rate and fractionation

In order to examine the dependency of the response of the films to dose rate, the films received 10 and 50 Gy dose at three different dose rates: 200, 400, and 600 MU/min. The experimental setup described above was employed for irradiating the films using 6 MV photon beams.

In order to evaluate the impact of fractionated beam delivery, several fractionation schemes were used, including 20 Gy delivered in 1, 2, and 4 fractions. Films were exposed to radiation every 24 h for multi-fraction sets. All fractionation sets started on the same date, and the films were scanned 48 h after the completion of the latest set, which corresponded to the 4-fraction regimen.

### 2.4 | Post-irradiation evolution of the optical density

In order to study the temporal changes in the OD following film irradiation, additional film samples were irradiated at 10 and 50 Gy using the 6 MV beam and subsequently scanned at varying intervals ranging from hours to days. The films were scanned every 3 h within the first 12-h window upon irradiation. Subsequently, the scanning frequency transitioned to every 12 h during the following days. As time progressed, the scanning intervals were further extended to span several days, gradually increasing in duration.

In order to quantify the temporal change in the Net OD of the films, we used a double (Equation 5) and triple (Equation 6) exponential model to fit the data.

(5) $$\text{Net}\;\mathrm{OD}(t)=\text{Net}\;OD_{\infty }-\left(C_{1}e^{-\frac{t}{T_{1}}}+C_{2}e^{-\frac{t}{T_{2}}}\right)$$

(6) $$\text{Net}\;\mathrm{OD}(t)=\text{Net}\;OD_{\infty }-\left(C_{1}e^{-\frac{t}{T_{1}}}+C_{2}e^{-\frac{t}{T_{2}}}+C_{3}e^{-\frac{t}{T_{3}}}\right)$$

where Net OD_∞_ is the Net OD corresponding to the latest readout of the film’s net OD, and $C_{1},C_{2},C_{3},T_{1},T_{2}$, and $T_{3}$ are different fitting parameters in each case.

### 2.5 | Uncertainties in dose measurement

In order to evaluate the uncertainties in dose measurement using the OC-1 films, using the pixel values and Net OD values of the films for each beam type, the fitting parameters, *a*, *b*, and *n*, were obtained through Equation (4). The total uncertainty is expressed through Equation (7).^16^

(7) $$\sigma_{tot}=\sqrt{\sigma_{exp}^{2}+\sigma_{fit}^{2}}$$

where $\sigma_{exp}$ and $\sigma_{fit}$, respectively, represent the experimental and fitting uncertainties which are obtained through the following equations

(8) $$\sigma_{exp}(\%)=\frac{\left(a\;+\;n\cdot b\cdot Net\;{OD}^{n-1}\right)\cdot \sigma_{Net\;OD}}{D_{fit}}\times 100$$

(9) $$\sigma_{fit}(\%)=\frac{\sqrt{Net\;{OD}^{2}\cdot \sigma_{a}^{2}\;+\;Net\;{OD}^{2n}\cdot \sigma_{b}^{2}}}{D_{fit}}\times 100$$

where $\sigma_{\text{Net}\;\text{OD}}$ represents the uncertainty in the Net OD measurement for each dose level.^28^

(10) $$\sigma_{\text{Net}\;OD}=\frac{1}{ln\;10}\sqrt{{\left(\frac{\sigma_{0}}{P_{0}}\right)}^{2}+{\left(\frac{\sigma_{f}}{P_{f}}\right)}^{2}}$$

In Equation (10), $\sigma_{0}$ and $\sigma_{f}$ are the statistical uncertainty of pixel values in un-irradiated and irradiated films, respectively.

In order to study the impact of using a generic calibration curve for all beam types, we used the calibration curve obtained for the 6 MV beam, and by using the Net OD values of the films irradiated by other beam types, calculated the percent error in the measured dose through

(11) $$\Delta (\%)=\frac{D_{exp}\;-\;D_{\text{mes}}}{D_{exp}}\times 100$$

where $D_{exp}$ is the expected delivered dose and $D_{\text{mes}}$ is the measured dose by the films using the 6 MV calibration curve.

### 2.6 | Spectroscopic measurement

Absorption spectra of the films were measured using a spectrometer (WP-VIS-R-S-100, Wasatch Photonics, Durham, NC) over the 500–750 nm spectral range with 2.5 nm spectral resolution. A white light emitting diode (LED) was used as a light source to illuminate the samples. An iris aperture was used to define a circular illumination region with ~1 cm diameter over the film samples. Five spectra, each with 30 ms acquisition time, were taken for each sample. The net absorbance was measured using Equation (12).

(12) $$\text{Net}\;A={log}_{10}\frac{I_{\text{blank}}\;-\;I_{\text{dark}}}{I_{\text{exposed}}\;-\;I_{\text{dark}}}$$

where $I_{\text{blank}}$ and $I_{\text{exposed}}$ represent blank film and exposed film, respectively, and $I_{\text{dark}}$ represents the spectrometer’s reading with its entrance slit covered to represent the dark signal.

## 3 | RESULTS

### 3.1 | Dose–response analysis

**FIGURE 3:**
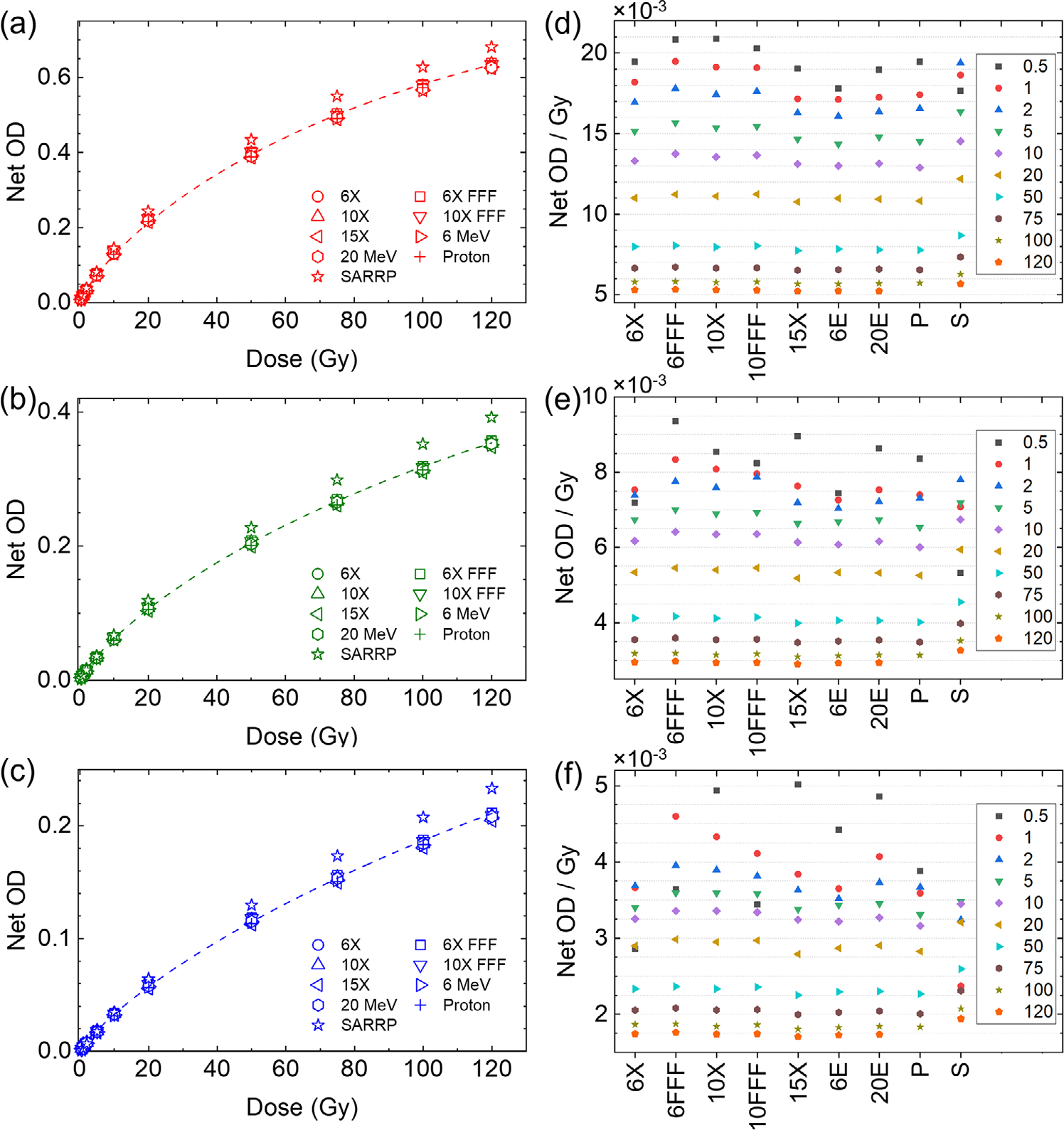
Net optical density of the (a) red, (b) green, and (c) blue color channels of the OC-1 films irradiated at various dose level using a variety of beam qualities. Corresponding net optical density per delivered dose is shown in panels (d–f).

Figure 3a–c show the net OD of the films irradiated using the linac, SARRP, and proton beams. The delivered dose levels were 0.5, 1, 2, 5, 10, 20, 50, 100, and 120 Gy, for which the corresponding MU (or the exposure time for the SARRP) was further corrected according to daily machine outputs on the day of film irradiation. Figure 3d–f show the Net OD per Gy for the red, green, and blue color channels, respectively. It can be seen that over 2 Gy is needed to reach a stable response. Beyond that, the dose response of the films to different beam qualities did not exhibit any significant dependence with an exception for the SARRP beam. In that case, an over-response was observed in all color channels. This discrepancy indicates that the 220 kVp beam exhibited a unique behavior compared to the other beam qualities evaluated in this work.

**FIGURE 4:**
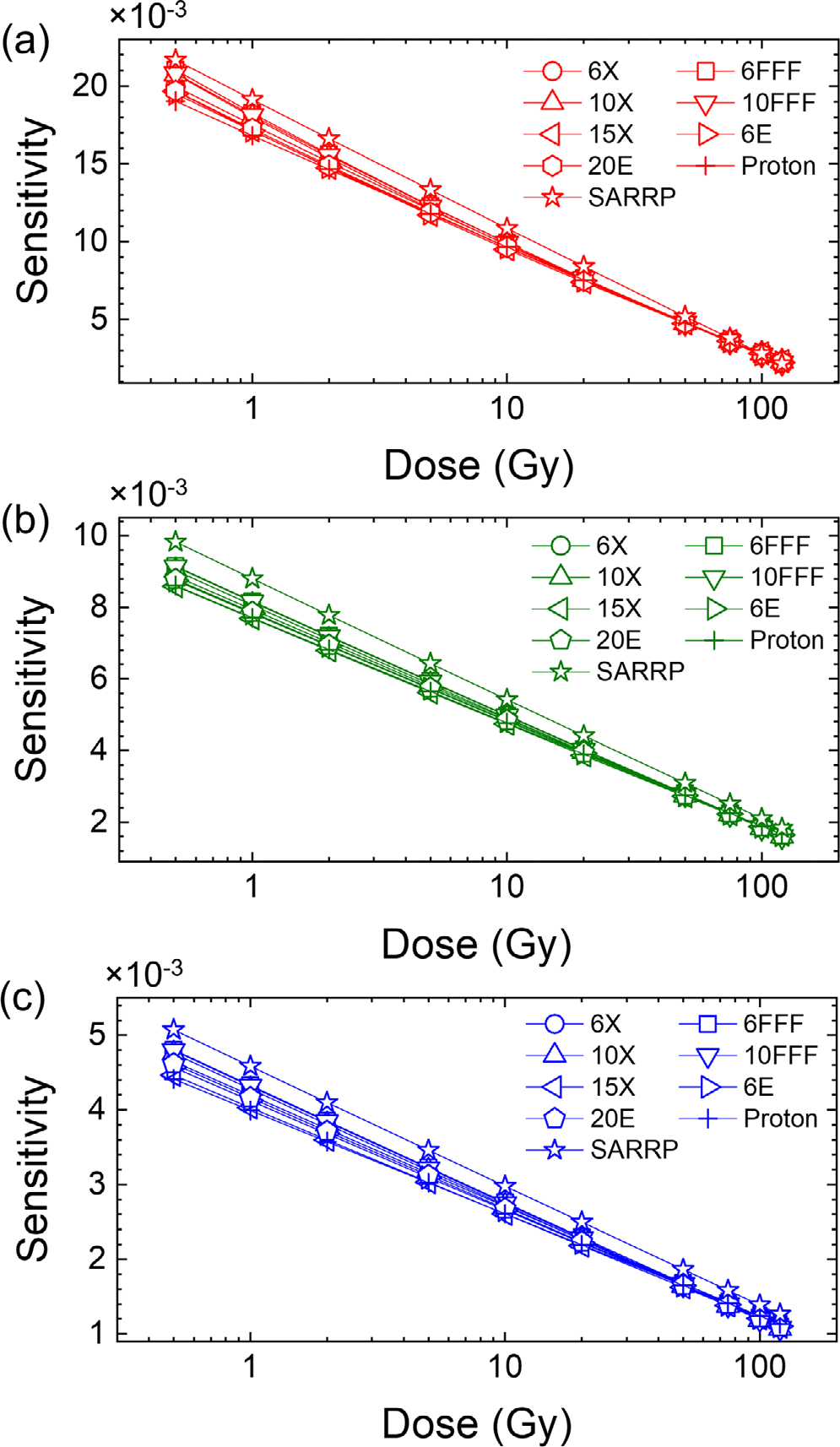
Sensitivity curves of OC-1 films irradiated with different beam types for (a) red, (b) green, and (c) blue color channels. 6X: 6MV x-ray, 6 FFF: 6 MV FFF x-ray, 10X: 10 MV x-ray, 10 FFF: 10 MV FFF x-ray, 15X: 15 MV x-ray, 6E: 6 MeV electron, 20 E: 20 MeV electron, proton: at 5 cm depth 227 MeV, SARRP: 220 kVp. SARRP, small animal radiation research platform.

To calculate the sensitivity of the films using Equation (3), curve fitting was performed for each beam quality and each color channel using Equation (2). The sensitivity analysis results are shown in Figure 4. It can be seen that the sensitivity decreases with dose; beyond ~5 Gy, all sensitivity curves converge except for the 220 kVp beam.

### 3.2 | Orientation dependency

**FIGURE 5:**
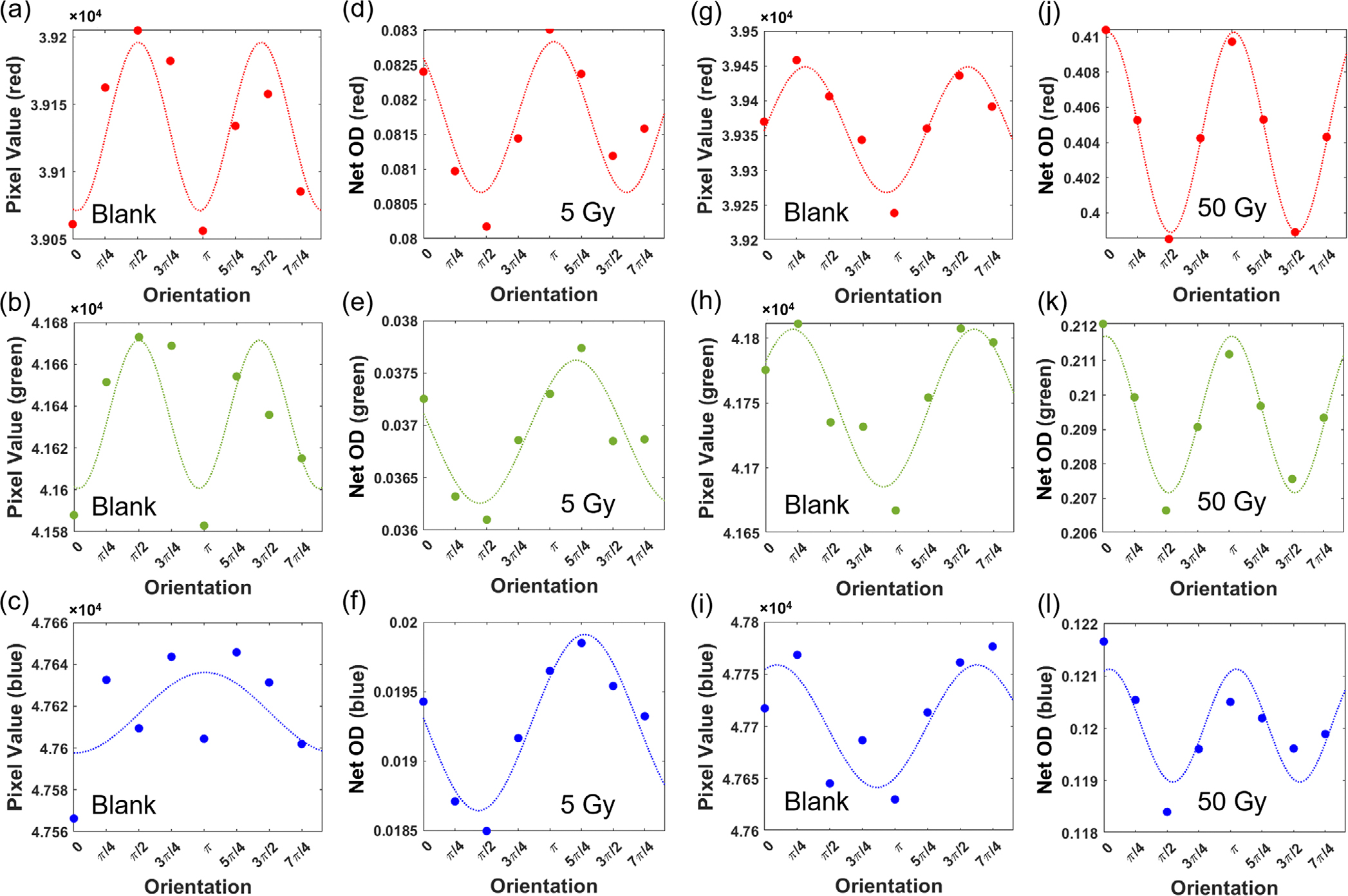
Orientation dependency of the OC-1 films. (a–c) Pixel values of an un-irradiated film measured at different orientation for red, green, and blue color channels, respectively. (d–f) The Net ODs of an irradiated film (5 Gy using 6 MV beam) at different film orientations for red, green, and blue color channels, respectively. (g–i) Pixel values of an un-irradiated film measured at different orientation for red, green, and blue color channels, respectively. (d–f) The Net ODs of an irradiated film (50 Gy using 6 MV beam) at different film orientations for red, green, and blue color channels, respectively. Orientations correspond to those indicated in Figure 1. OD, optical density.

Figure 5 shows the orientation dependency of the response of the films during scanning. For the irradiated films, the Net OD measured at different orientations was plotted. For the un-irradiated films, since obtaining a “reference” reflected image was not straightforward, the pixel values measured at different orientations were plotted as shown in Figure 5a–c and g–i. The corresponding films were irradiated by a 6 MV beam at 5 and 50 Gy dose and their Net ODs are displayed with respect to orientations for all color channels in Figure 5d–f and j–l, respectively. A squared sinusoidal fit to the data was performed. According to Malus’ law,^29^ the light intensity after passing a linear polarizer is *I* = *I*_0_ cos^2^*θ*, in which *I*_0_ is the light intensity in the absence of the polarizer and *θ* is the relative angle between the polarizer’s axis and the polarization direction of the light source. It can be seen that a sinusoidal pattern exists in the data, indicating the nature of the difference is due to polarization related effects. It is crucial to maintain a consistent orientation when scanning the films to ensure accurate and reliable results. For this purpose, all film measurements in this work were scanned in orientation 1 (i.e. landscape orientation of the film sheet before cuting it into smaller samples), as depicted in Figure 1.

### 3.3 | Dependency on dose rate and fractionation

**FIGURE 6:**
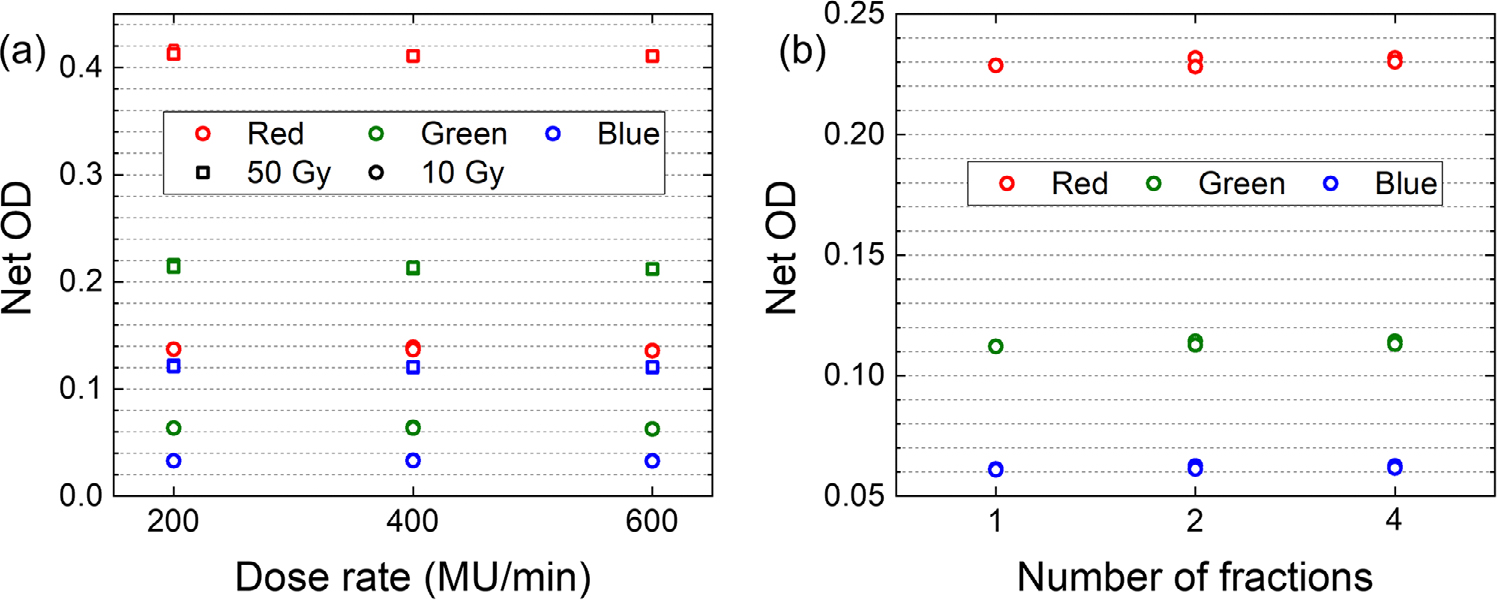
(a) Net OD versus dose rate measured for red, green, and blue color channels for films irradiated with 10 and 50 Gy using a 6 MV beam at three dose rates of 200, 400, and 600 MU/min. Each dose level and dose rate was repeated for three film samples. (b) Films were irradiated with a total dose of 20 Gy in three different fractionation schemes: 1 fraction, 2 fractions, and 4 fractions. 6 MV beam was used to irradiate the films at 24 h intervals. Each fractionation scheme was repeated with three film samples. Error bars are smaller than the symbols. OD, optical density.

Figure 6a shows the Net OD of the films irradiated using 6 MV beams at dose levels of 10 and 50 Gy, with three different dose rates of 200, 400, and 600 MU/min. The analysis revealed no significant dependence between the dose rate and the film’s response for all color channels.

In order to evaluate the impact of fractionation, the films underwent daily (24 h interval) exposures to a total dose of 20 Gy using three different fractionation schemes: 1 fraction (20 Gy × 1 fx), 2 fractions (10 Gy × 2), and 4 fractions (5 Gy × 4). All fractionation schemes started on the same day (day 1) and were completed on consequent days: day 1, day 2, and day 4, respectively. All films were scanned on day 6, 48 h after the 4^th^ fraction scheme was completed. The analysis, as depicted in Figure 6b, shows no significant correlation between the fractionation and the film’s response which indicates that variations in the fractionation regimen did not have a notable impact on the observed film response.

### 3.4 | Post-irradiation response evolution

**FIGURE 7:**
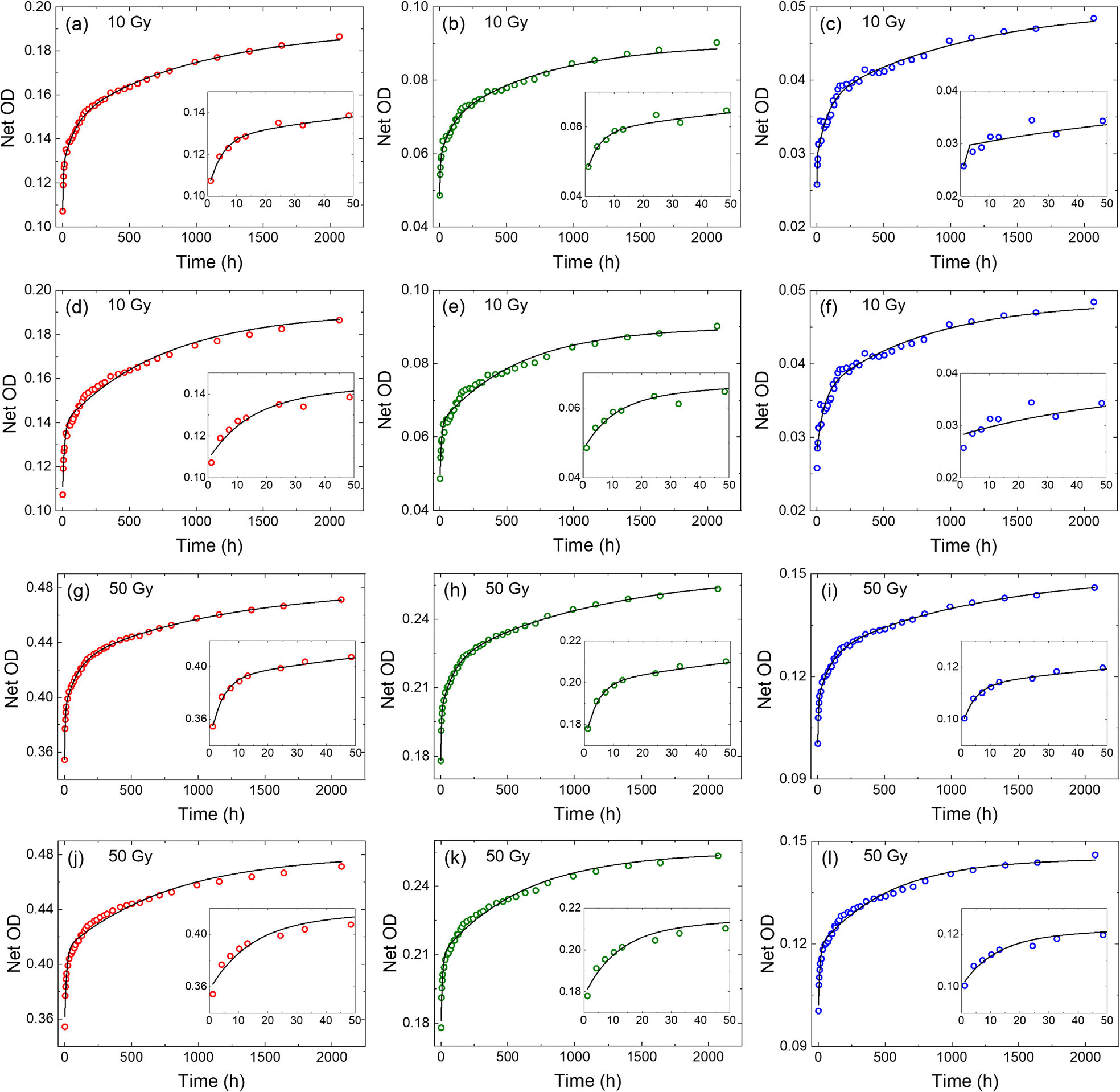
Temporal changes in the Net OD following film irradiation. Net ODs of three color channels are shown for films irradiated with (a–f) 10 Gy and (g–l) 50 Gy using a 6 MV beam. The three-exponential model was fitted in panels (a–c) and (g–i). The two-exponential model was fitted in panels (d–f) and (j–l). The insets show changes in the Net OD within the first 48 h post-irradiation. OD, optical density.

The temporal change in the response of the films following the film irradiation was measured. Films were irradiated by 6 MV beams and subsequently scanned at varying intervals ranging from hours to days. Two dose levels (10 and 50 Gy) were evaluated. In Figure 7a–f and g–l, the change in Net OD for all color channels is presented for films irradiated with 10 and 50 Gy dose, respectively. Approximately 30% (15%), 33% (18%), and ~33% (19%) increase in the Net OD of the red, green, and blue color channel, respectively, can be seen during the first 48 h post-irradiation for the film irradiated with 10 Gy (50 Gy). After that a steady growth of Net OD at a lower slope was observed during the 90-days period of the study: ~35% (40%) for the red, ~39% (40%) for the green, and ~41% (40%) for the blue channel in the 10 Gy (50 Gy) irradiated film. This indicates a total change in the Net OD of approximately 74% (33%), 86% (42%), and 88% (45%) for the red, green, and blue color channels, respectively, for the 10 Gy (50 Gy) irradiated film during the 3-months period post-irradiation. These plots confirm the importance of scanning the calibration films and test films at the same time interval post-irradiation.

In order to quantify the temporal change in Net OD, we used the two-exponential (Equation 5) and three-exponential (Equation 6) models to fit the data. The resultants fits are presented in Figure 7. It can be seen that the fits can predict the Net OD with ~1% error for the red and green channels. The fitting parameters appear to depend on the dose and color channel as tabulated in Table 1. It can be seen that the three exponential model led to smaller error compared to the double-exponential model.

### 3.5 | Uncertainty and error analysis

**FIGURE 8:**
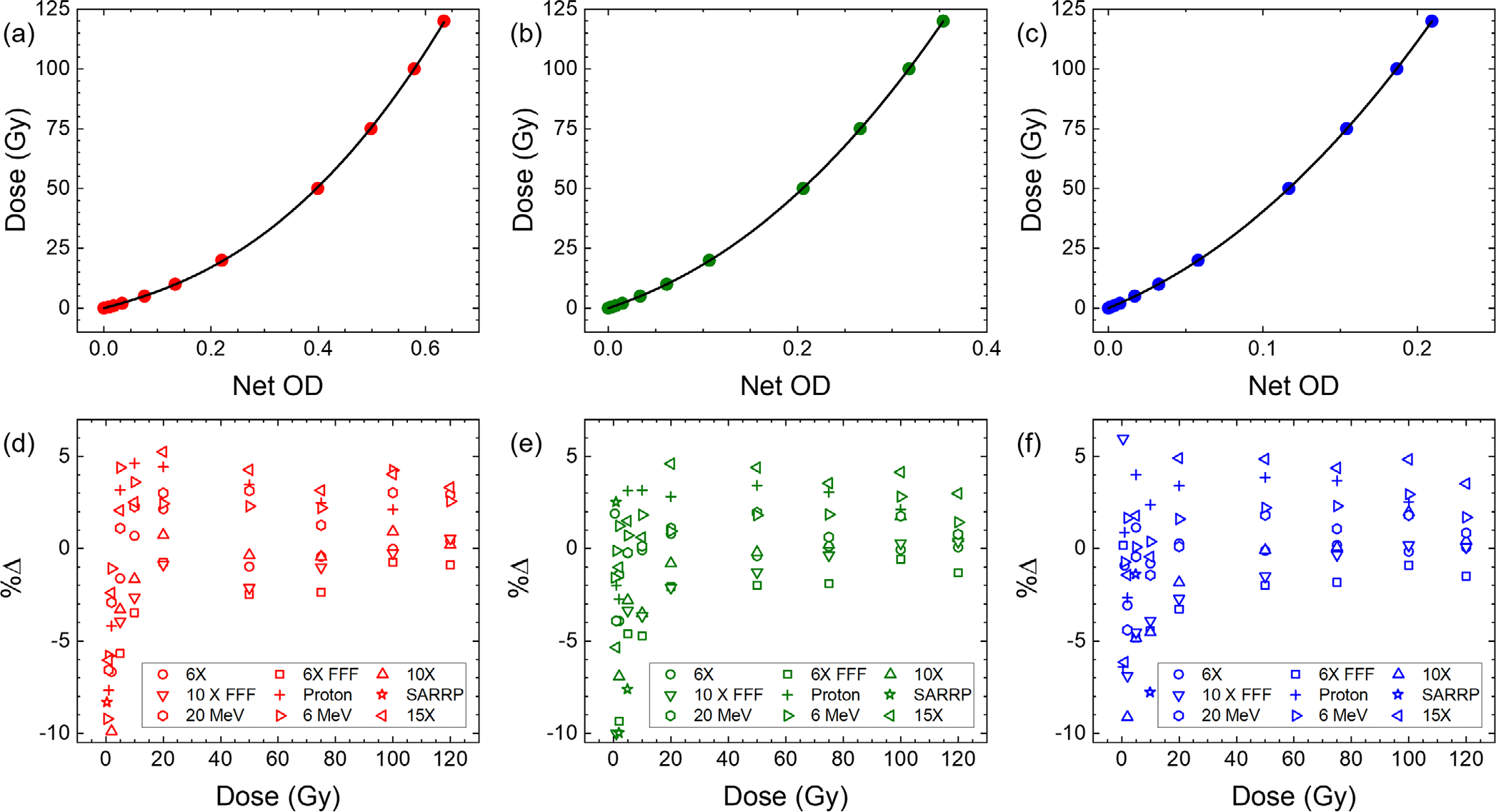
Calibration curves for the (a) red, (b) green, and (c) blue color channels for the films irradiated by 6 MV photons. Percent difference between the intended delivered dose and measured dose by using the 6 MV calibration curve and Net OD of the (c) red, (d) green, and (f) blue color channel for all beam types studied in this work. For visualization purpose, panels (d–f) are set to show values between −10% and 6%. For doses ≥5 Gy, the percent difference is always between ±6% for all radiation types other than the SARRP. The fitting parameters of the 6 MV films were used to evaluate the percent difference in dose measurement for other films. OD, optical density; SARRP, small animal radiation research platform.

Figure 8a–c show the calibration curves for the red, green, and blue color channel, respectively, for the films irradiated using the 6 MV beam. The fitting parameters for the calibration curves for all beam types studied in this work are tabulated in Table 2.

The percentage difference between the expected delivered dose using all beam types and the measured dose using the calibration curve of the 6 MV beam is plotted in Figure 8d–f for the red, green, and blue color channel, respectively. For all color channels and doses ≥5 Gy, the percent difference between the intended dose and measured dose using the 6 MV calibration curve (except for the SARRP) lies within ±~5% with majority of them within ±3% of the expected value. At lower doses the difference is increases due to instability of the response. For visualization purpose, panels (d–f) in Figure 8 are set to show values between −10% and 6%.

**FIGURE 9:**
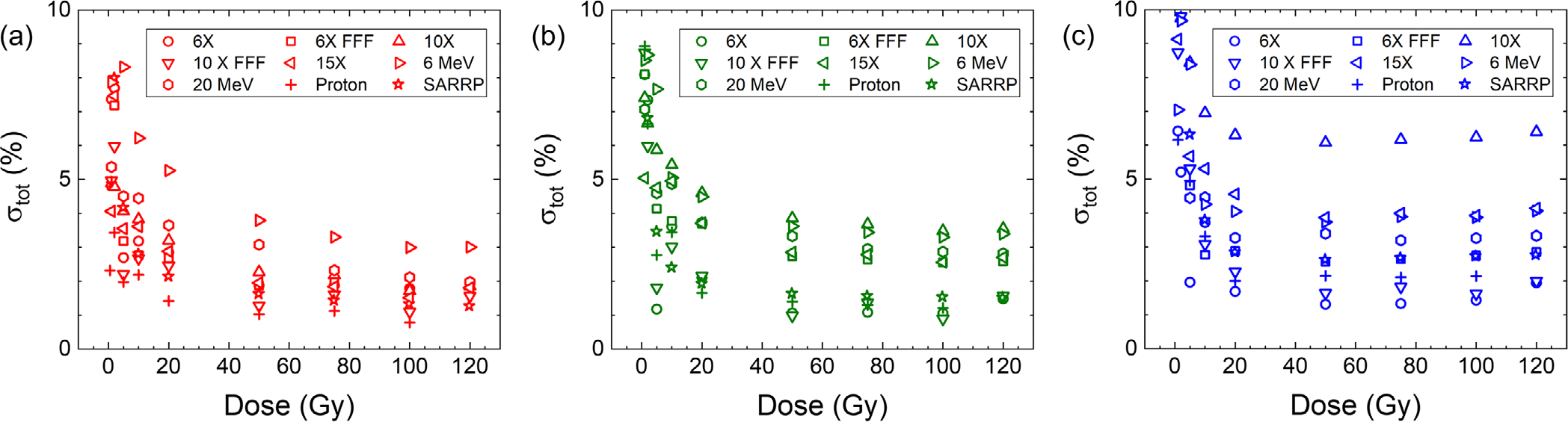
(a–c) Associated total uncertainty calculated at each dose level and radiation type for red, green, and blue color channels, respectively. For visualization purpose, values up to 10% are shown.

The total uncertainty, *σ*_tot_, in dose measurement using each calibration curve and at each dose level calculated according to the procedure described in Section 2.5 for the red, green, and blue color is presented in Figure 9a–c.

### 3.6 | Spectroscopic measurement

**FIGURE 10:**
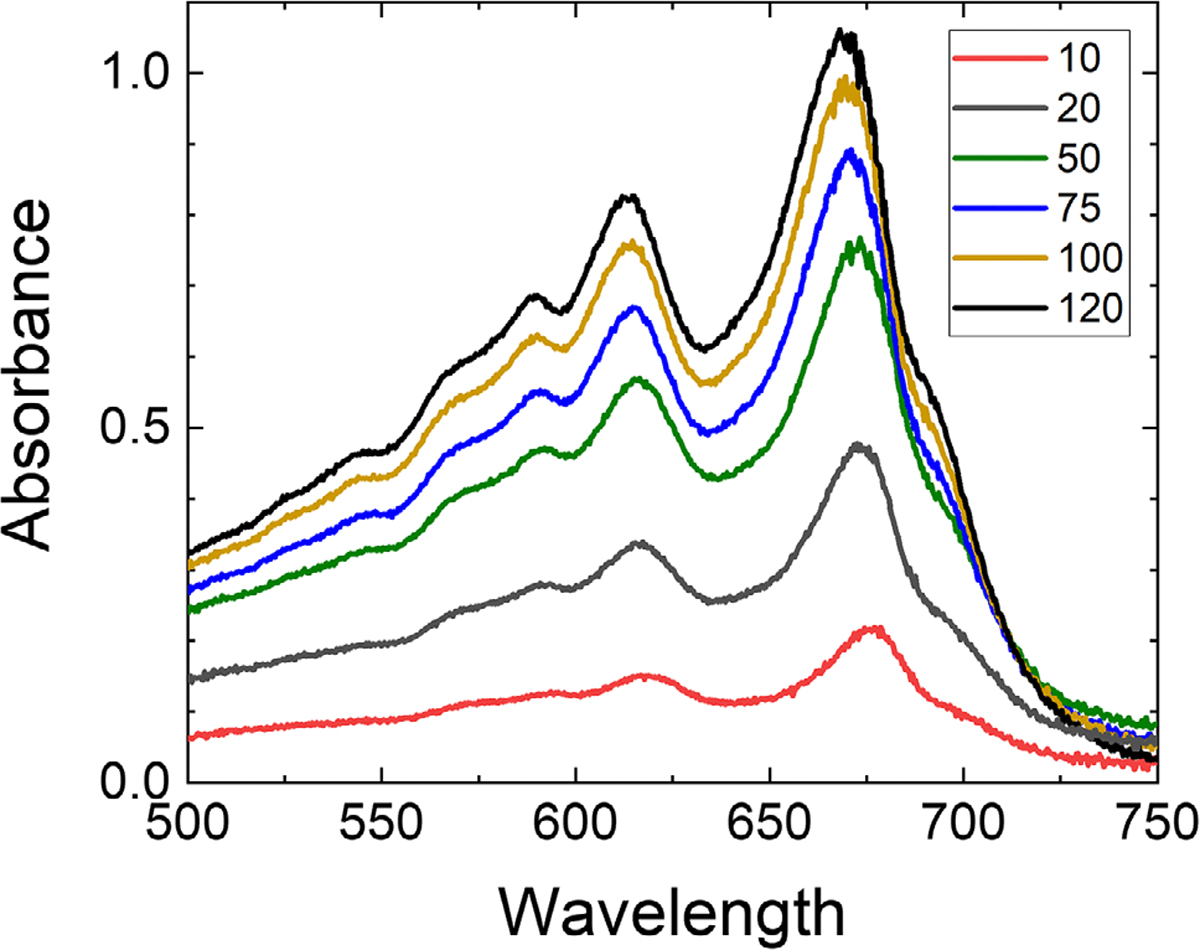
Net absorbance spectra of films irradiated at different dose levels (10–120 Gy) using the SARRP. SARRP, small animal radiation research platform.

Figure 10 shows a series of absorption spectra of the films irradiated at different dose levels using the SARRP. The primary absorption peak was observed at ~670 nm. The secondary and tertiary absorption peaks were observed at ~615 and ~590 nm. It can be seen that the position of the peaks are slightly blue-shifted as the dose increases. No significant change in the location of the absorption peaks and overall spectral shapes (except for their amplitude) were noted with respect to the radiation type.

## 4 | DISCUSSION

The response of the OC-1 films, irradiated at >2 Gy, did not show significant energy dependency in megavoltage photon and electron beams, as well as in the plateau region of a proton beam used in this work in agreement with a previous study.^22^ However, at the 220 kVp energy, an over-response by 10% was observed in the Net OD of the films compared to the response of the films to the megavoltage beams. RCFs are known to have LET-dependency in high-LET fields.^30^ However, our observation with the OC-1 films showed an unexpected behavior compared to EBT3 (and EBT-XD) models in which an under-response to kV beams has been reported. For example, ~10% under-response has been reported for EBT3 films.^31^ In another study, for EBT3 and EBT-XD 10%–15% under-response was found at kV beams.^32^ The over-response in OC-1 films to kV beams may be related to their material composition and warrants further investigation at various kVp conditions. We noted a variation in the response of the films irradiated at low doses (0.5 and 1 Gy) indicating that caution must be exercised when using a generic calibration curve for these films to measure low doses.

Our proton irradiation was performed at the plateau region (5 cm) where the LET is not high. As such, we did not observe any significant beam type difference between the films irradiated by proton, photon, and electron beams. It would be interesting to perform the experiments at the Bragg peak as well to evaluate their quenching.

We did not observe a difference in the OD of the films when the same dose was delivered through several fractions at 24 h time intervals. The observed results showed the same behavior reported for EBT3 and EBT-XD films.^33,34^

It had been reported that OC-1 films did not show dose rate dependency when irradiated by proton beams at ultra-high dose rates (15 Gy/min up to 4.5 × 10^5^ Gy/min) proton beams produced by an isochronous cyclotron.^23^ Here, we irradiated the films at clinical dose rates of ~2–6 Gy/min using a commonly used 6 MV photon beam and did not observe a dose rate dependency.

Previous studies have shown that the Net OD of RCFs grow over time post-irradiation.^22,35,36^ Here, temporal growth of the post-irradiation OD was observed in OC-1 films as well. This behavior has also been observed in EBT3 and EBT-XD films.^34^ The initial rate of the growth in the Net OD is comparable between these models with ~20% growth in first 48 h followed by a gradual increase in OD beyond that. Previous work reported ~1% change between Net OD measured at 24 and 120 h for the EBT3 and EBT-XD films. It has been reported that the red-channel OD in OC-1 films irradiated at 10 Gy dose increased ~10% over 24 h and ~18% over 10 days postirradiation.^22^ Here, we observed 10%–15% growth in the Net OD between 24 and 120 h. More specifically, for the film irradiated at 10 Gy dose, we observed that the Net OD increased by ~20% over 24 h and by ~40% over 10 days post-irradiation. It appears that the response of OC-1 model constantly grows over time at a higher rate compared to EBT3 and EBT-XD models.

We observed ~3% change in the Net OD depending on the orientation of the film with respect to the scanner which can translate to ~10% change in the measured dose. The observed orientation dependency in the measured OD can be explained by a model based on the existence of a preferential polarization direction in the films. It is well known that parallel nano-structured can act as a polarizer, absorbing the component of the electric field parallel to the stripes. Our observation that the OD dropped under certain orientations is in general agreement with that assumption. In order to carefully examine that one can perform scanning electron microscopy (SEM) of the films. The needle shaped crystal constituents of the film are reminiscent to those in polaroid polarizers.^29^ A preferred elongation of the monomers acts like a polarizer in which the direction of the polarization of the transmitted light is perpendicular to the direction of the monomers.

Our spectroscopic study revealed that the spectral position of the absorption peaks of the OC-1 films, located at ~670, ~615, and ~590 nm, are blue-shifted with dose. In EBT3, EBT-XD, and MD-V3 film models, the primary and secondary absorption peaks are located at ~634 and ~584 nm.^21,33,37^ In HD-V2 films model, the absorption peaks are located at ~670 and ~615 nm.^20,38^ For the MD-V3 model, it has been reported that primary (~634 nm) and secondary (~584 nm) peaks are red-shifted with dose.^21^ For the HD-V2 model (Ashland) model, it has been reported that primary (~634 nm) and secondary (~619 nm) peaks are blue-shifted with dose.^20,38^ This phenomenon can be linked to the size of the polymers and radiation-induced changes in the microstructure of the film.^39^

## 5 | CONCLUSION

OC-1 films appeared to be dose-rate independent within the clinical dose rates studied in this work. The energy dependency between different megavoltage photon and electron beams was insignificant for films irradiated at ≥ 5 Gy. However, ~10% over-response was noted in the response of the films irradiated by 220 kVp x-rays compared to those irradiated by MV x-rays. No impact of fractionated delivery with 24 h delay between exposures was observed. Approximately 3% orientation dependency in the measured OD was noted which is a manifestation of modulation of light polarization by such films. The response of the films continuously grew over time following a double or triple exponential model.

## Figures and Tables

**TABLE 1:** Fitting parameters obtained through fitting the net optical density (OD) versus time considering 3-component and 2-component models.

	10 Gy (R)	50 Gy (R)	10 Gy (G)	50 Gy (G)	10 Gy (B)	50 Gy (B)

Equation 6						
*C*_1_	0.0429 ± 0.0011	0.0557 ± 0.001	0.0222 ± 0.0013	0.04 ± 0.0007	0.0129 ± 0.0008	0.024 ± 0.0006
*C*_2_	0.0199 ± 0.0012	0.0342 ± 0.001	0.0096 ± 0.0013	0.0211 ± 0.0008	0.0077 ± 0.0008	0.0132 ± 0.0007
*C*_3_	0.0255 ± 0.0015	0.0475 ± 0.0015	0.0127 ± 0.0016	0.0281 ± 0.0011	236.95	0.016 ± 0.0009
*T*_1_	966.67 ± 39.24	1128.87 ± 33.50	758.54 ± 61.55	1110.27 ± 33.29	1105.91 ± 130.69	1141.1 ± 49.24
*T*_2_	86.86 ± 11.03	90.47 ± 5.98	81.148 ± 23.41	88.46 ± 6.93	77.32 ± 17.67	88.85 ± 9.73
*T*_3_	4.496 ± 0.61	4.134 ± 0.29	4.33 ± 1.22	4.04 ± 0.36	0.101	4.29 ± 0.57
Net OD_∞_	0.19	0.48	0.09	0.26	0.05	0.15
% Δ	0.43	0.13	0.93	0.21	1.71	0.34
Equation 5						
*C*_1_	0.0508 ± 0.0009	0.0692 ± 0.002	0.026 ± 0.0006	0.0451 ± 0.0009	0.0125 ± 0.0009	0.026 ± 0.0006
*C*_2_	0.0304 ± 0.0022	0.0532 ± 0.004	0.0155 ± 0.0015	0.0319 ± 0.0022	0.0078 ± 0.0009	0.0188 ± 0.0014
*T*_1_	751.48 ± 37.33	797.82 ± 48.14	597.49 ± 31.78	624.87 ± 29.58	800.72 ± 100.15	528.68 ± 26.38
*T*_2_	14.408 ± 2.38	14.04 ± 2.20	10.818 ± 2.068	11.28 ± 1.59	52.04 ± 14.13	11.04 ± 1.645
Net OD_∞_	0.19	0.48	0.09	0.255	0.0485	0.145
% Δ	1.27	0.76	1.48	0.75	2.07	0.79

**TABLE 2:** Fitting parameters for OC-1 films irradiated by different beam types.

Beam type	Red	Green	Blue

6 MV	*a*: 61.067 ± 1.79 *b*: 244.306 ± 5.47 *n*: 2.434 ± 0.053 *R^2^*: 1.0000	*a*: 135.677 ± 1.66 *b*: 681.75 ± 10.92 *n*: 2.166 ± 0.020 *R^2^*: 1.0000	*a*: 272.657 ± 3.67 *b*: 1732.78 ± 42.18 *n*: 2.120 ± 0.022 *R^2^*: 1.0000
6 MV FFF	*a*: 57.484 ± 1.90 *b*: 244.443 ± 4.73 *n*: 2.411 ± 0.054 *R^2^*: 1.0000	*a*: 127.363 ± 5.61 *b*: 680.001 ± 20.55 *n*: 2.141 ± 0.050 *R^2^*: 0.9999	*a*: 251.301 ± 7.76 *b*: 1645.941 ± 71.86 *n*: 2.059 ± 0.042 *R^2^*: 0.9999
10 MV	*a*: 60.035 ± 2.62 *b*: 248.239 ± 2.91 *n*: 2.438 ± 0.051 *R^2^*: 0.9999	*a*: 125.872 ± 7.89 *b*: 685.152 ± 27.14 *n*: 2.111 ± 0.067 *R^2^*: 0.9999	*a*: 242.547 ± 19.19 *b*: 1595.757 ± 147.57 *n*: 1.997 ± 0.091 *R^2^*: 0.9999
10 MV FFF	*a*: 61.928 ± 1.43 *b*: 257.175 ± 1.94 *n*: 2.546 ± 0.036 *R^2^*: 0.9999	*a*: 129.282 ± 1.79 *b*: 713.011 ± 7.37 *n*: 2.172 ± 0.016 *R^2^*: 1.0000	*a*: 254.451 ± 4.43 *b*: 1745.485 ± 45.58 *n*: 2.085 ± 0.024 *R^2^*: 0.9999
15 MV	*a*: 62.729 ± 2.38 *b*: 250.02 ± 2.59 *n*: 2.401 ± 0.045 *R^2^*: 0.9999	*a*: 130.806 ± 6.22 *b*: 650.142 ± 18.21 *n*: 2.048 ± 0.049 *R^2^*: 0.9999	*a* :257.131 ± 13.13 *b*: 1477.178 ± 86.23 *n*: 1.944 ± 0.058 *R^2^*: 0.9999
6 MeV	*a*: 62.024 ± 4.66 *b*: 253.63 ± 5.28 *n*: 2.431 ± 0.089 *R^2^*: 0.9998	*a*: 130.431 ± 7.76 *b*: 665.486 ± 25.13 *n*: 2.090 ± 0.065 *R^2^*: 0.9999	*a*: 259.193 ± 11.98 *b*: 1558.403 ± 94.48 *n*: 2.002 ± 0.059 *R^2^*: 0.9999
20 MeV	*a*: 64.731 ± 3.14 *b*: 256.029 ± 4.12 *n*: 2.501 ± 0.066 *R^2^*: 0.9999	*a*: 132.399 ± 6.55 *b*: 662.212 ± 22.23 *n*: 2.107 ± 0.057 *R^2^*: 0.9999	*a*:255.258 ± 10.04 *b*: 1543.538 ± 77.84 *n*: 1.999 ± 0.05 *R^2^*: 0.9999
SARRP	*a*: 65.836 ± 4.43 *b*: 229.679 ± 7.88 *n*: 2.920 ± 0.148 *R^2^*: 0.9998	*a*: 134.045 ± 5.65 *b*: 604.733 ± 26.97 *n*: 2.339 ± 0.075 *R^2^*: 0.9999	*a*: 271.674 ± 8.58 *b*: 1699.397 ± 146.22 *n*: 2.334 ± 0.079 *R^2^*: 0.9999
Proton	*a*: 62.783 ± 1.04 *b*: 240.257 ± 1.58 *n*: 2.374 ± 0.023 *R^2^*: 0.9999	*a*: 131.962 ± 2.47 *b*: 629.222 ± 9.68 *n*: 2.046 ± 0.023 *R^2^*: 0.9999	*a*: 263.931 ± 5.74 *b*: 1432.990 ± 50.33 *n*: 1.958 ± 0.031 *R^2^*: 0.9999

SARRP, small animal radiation research platform.

## Data Availability

The datasets used during the current study are available from the corresponding author on reasonable request.
